# Agreement between a new optical low coherence reflectometry biometer and an anterior segment optical coherence tomographer

**DOI:** 10.1186/s40662-023-00330-9

**Published:** 2023-02-25

**Authors:** Yiran Wang, Ting Wan, Luze Liu, Yuyuan Xue, Xinyao Chen, Giacomo Savini, Domenico Schiano-Lomoriello, Xingtao Zhou, Jinjin Yu, Jinhai Huang

**Affiliations:** 1 Eye Institute and Department of Ophthalmology, Institute for Medical and Engineering Innovation, Eye & ENT Hospital, Fudan University; NHC Key Laboratory of Myopia (Fudan University), Key Laboratory of Myopia, Chinese Academy of Medical Sciences, No.19 Baoqing Road, Xuhui District, Shanghai, 200031 China; 2 Shanghai Research Center of Ophthalmology and Optometry, Shanghai, China; 3 Eye Hospital and School of Ophthalmology and Optometry, Wenzhou Medical University, Wenzhou, Zhejiang China; 4 G.B. Bietti Foundation I.R.C.C.S., Rome, Italy

**Keywords:** SW-9000, MS-39, Agreement, Ocular biometric parameters, Optical biometry

## Abstract

**Background:**

To assess agreement of measurements between a new optical low coherence reflectometry (OLCR) biometer (SW-9000, Suoer, Tianjin, China) and a spectral-domain optical coherence tomographer (SD-OCT)/Placido topographer (MS-39, CSO, Florence, Italy) in healthy subjects.

**Methods:**

A total of 66 right eyes from 66 healthy subjects were enrolled in this prospective study. Three consecutive measurements were randomly obtained with both devices by the same experienced operator to assess agreement. Bland-Altman plots and 95% limits of agreement (LoA) were used to verify the agreement between the devices. Results are presented as mean ± standard deviation (SD).

**Results:**

The SD-OCT/Placido tomographer showed high agreement with the OLCR biometer for all parameters included in this study. The mean differences of central corneal thickness (CCT), anterior chamber depth (ACD), aqueous depth (AQD), mean keratometry (Km) and corneal diameter (CD) were 2.21 ± 2.67 μm (*P* < 0.001), − 0.10 ± 0.03 mm (*P* < 0.001), − 0.10 ± 0.04 mm (*P* < 0.001), − 0.01 ± 0.22 D (*P* = 0.773) and 0.20 ± 0.16 mm (*P* < 0.001), respectively. This implies that the inter-device difference in Km was not statistically significant, while the differences in CCT, ACD, AQD, CD were statistically but not clinically significant. The 95% LoAs of CCT, ACD, AQD, Km and CD were − 3.01 to 7.44 μm, − 0.16 to − 0.05 mm, − 0.18 to − 0.03 mm, − 0.45 to 0.43 D, and − 0.12 to 0.51 mm, respectively.

**Conclusions:**

For CCT, ACD, AQD, Km, and CD in healthy subjects, the new OLCR biometer has high agreement with the SD-OCT/Placido tomographer and can be used interchangeably due to the narrow range of 95% LoAs.

## Background

Biometry is indispensable for the diagnosis and treatment of ophthalmic diseases. Keratometry (K), axial length (AL), and anterior chamber depth (ACD) are important variables in the initial intraocular lens (IOL) calculation formulas [1–3]. Newer algorithms such as the Holladay 2 and Barrett Universal II formulas also use corneal diameter (CD) and lens thickness (LT) to improve accuracy [4, 5]. Olsen formula optionally included central corneal thickness (CCT) in addition to LT. Similarly, the Kane and Emmetropia Verifying Optical (EVO) formulas also consider CCT as an optional parameter for IOL power calculation [6]. Furthermore, ACD and aqueous depth (AQD) are crucial parameters for calculating the power of phakic IOLs as well as for selecting candidates for phakic IOLs [1, 7, 8]. The K value and CD are important references for contact lens selection or fitting [9, 10]. CCT is crucial for corneal refractive surgery [11]. An early diagnosis of keratoconus and glaucoma also depends on anterior segment measurements [12, 13].

The SW-9000 (Suoer, version 1.0.00.R, Tianjin, China) is an optical biometer, whereas the MS-39 (CSO, version 4.0.0.57, Florence, Italy) is an anterior segment optical coherence tomographer (AS-OCT) combined to a Placido disc topographer. The SW-9000 measures CCT, K, ACD, CD, AL, and LT by applying optical low coherence reflectometry (OLCR). ACD and AQD are defined as the distance from the corneal epithelium and endothelium to the anterior surface of the crystalline lens, respectively. Hence, AQD is numerically equal to CCT minus ACD. The MS-39, which has been the first device to integrate spectral-domain OCT (SD-OCT) and Placido disc corneal topography, can also obtain CCT, K, ACD, AQD and CD measurements [14].

However, there are no studies on the biometric measurements of SW-9000. Furthermore, the MS-39 has never been compared to an OLCR instrument. Therefore, the purpose of this study was to evaluate the accuracy of SW-9000 and agreement with MS-39 in measuring the fundamental anterior segment parameters: CCT, mean keratometry (Km), ACD, AQD and CD.

## Methods

### Subjects

This prospective study, conducted in accordance with the principles of the Declaration of Helsinki, was approved by the Ethics Committee of the Eye and ENT Hospital of Fudan University (Shanghai, China). Enrolled subjects were informed in advance about the objective of the study and signed an informed consent form. Prior to any measurement, all eyes underwent detailed ophthalmic examination without pupillary dilation, including subjective refraction, slit-lamp microscopy, ophthalmoscopy, and non-contact tonometer (NCT), in order to exclude any abnormal eyes. The specific exclusion criteria were as follows: ocular or corneal diseases other than ametropia, a history of previous corneal or intraocular surgery which could modify the measurement [15, 16], and a recent history of wearing contact lens (four weeks for rigid gas permeable contact lens and two weeks for soft contact lens[17]), and difficulty in cooperation during the study.

### Instruments

#### SW-9000 OLCR biometer

The SW-9000 OLCR biometer adopts a superluminescent light emitting diode (SLED, 840 ± 10 nm) to capture eight different measurements in less than 5 s; these include CCT, ACD, LT, AL, K, CD, and pupil diameter (PD). By subtracting ACD from CCT, the value of AQD is obtained. Axial data are obtained by OLCR from the optical path distance from the anterior surface of the cornea to the retinal pigment epithelium. CD and PD are gained through anterior segment image. The instrument recorded the reflections of six points projected on the anterior surface of the cornea, and the K value was obtained by analysis.

#### MS-39 SD-OCT/Placido tomographer

The MS-39 SD-OCT/Placido tomographer integrates SD-OCT and a Placido-disk corneal topographer into a single device to acquire anterior segment measurements. Employing a SLED light source of 845 nm, it provides images with higher resolution than any Scheimpflug camera. A total of 25 section images, one keratoscopy, and one iris front image can be obtained with each scan, which takes only about a second. High-resolution tomography of the anterior segment provides corneal and anterior chamber parameters. In addition, Placido-disc technology provides reliable measurements of the anterior surface of the cornea based on the law of reflective optics. Hence, it provides comprehensive information on the anterior segment.

### Measurement technique

For assessing the agreement between SW-9000 and MS-39, all participants were measured by one well-trained operator three times in a random order. All measurements were taken in a dimly lit room between 9 a.m. and 5 p.m. to minimize diurnal ocular changes. Before each measurement, participants were told to blink so as to obtain a smooth tear film and then fixate on a built-in fixation with each device. They were re-positioned at the instrument before the next scan was acquired. Only the right eye was evaluated, and only qualified scans indicated by the instrument were used for analysis; otherwise, the procedure was repeated. The images gained by SW-9000 are considered good when all data results are obtained in one measurement and no “exclamation marks” appear. Images taken by MS-39 were considered acceptable if a “green check mark” appeared on machine interpretation and a manual review showed adequate corneal exposure. The entire procedure lasted for less than 30 min.

### Statistical analysis

Statistical analysis was conducted by SPSS software (V.21.0; IBM Corp., New York, USA). A paired t-test was used to compare the measurements by the two devices, and Kolmogorov-Smirnov test was used to assess the normality of the data (*P* > 0.05). Pearson’s correlation analysis and linear regression were performed for the parameters measured by the two instruments. Agreement between SW-9000 and MS-39 was evaluated through Bland-Altman plots and the 95% limits of agreement (LoA) which were performed using MedCalc software (V.19.8; MedCalc Software Ltd., Ostend, Belgium). The 95% LoA was calculated as the mean difference ± 1.96 SD [18]. Sample size was calculated by the following formula: $n={(\frac{{Z}_{1-\frac{a}{2}}\times \sqrt{p\left(1-p\right)}}{\updelta })}^{2}$, Z_1-a/2_ is 1.96, p stands for specificity or sensitivity, δ represents allowable error. Results were presented as mean ± standard deviation (SD). *P* < 0.05 was considered statistically significant.

## Results

A total of 66 right eyes from 66 healthy subjects were enrolled in this study. Among them, 41 were females and 25 were males. The mean age was 27.57 years ± 5.70 (SD) (range, 18 to 38 years). The spherical refraction was − 4.59 ± 2.06 (range, − 1.50 to − 8.00) diopters (D) and cylinder was − 0.75 ± 0.68 (range, − 0.25 to − 2.75) D. The equivalent spherical power ± SD was − 4.88 ± 1.90 (range, − 1.50 to − 10.38) D. The Pearson’s correlation coefficients of CCT, ACD, AQD, Km and CD were respectively 0.996, 0.989, 0.989, 0.972, 0.892 (all *P* < 0.000), indicating a high correlation.

Table 1 summarizes the values of parameters measured by SW-9000 and MS-39 represented as mean ± SD, including CCT, ACD, AQD, Km and CD. Table 2 indicates the differences and agreement between MS-39 and SW-9000. Except for Km, the paired t-test of the two instruments showed statistically significant differences for all measurement (*P* < 0.001). Measurements by the MS-39 were slightly higher than those by the SW-9000 for ACD, AQD and Km. The results were the converse for CCT and CD.

**Table 1:** Biometric measurements provided by the SW-9000 and MS-39

Parameter	SW-9000			MS-39		
	Mean ± SD	Minimum	Maximum	Mean ± SD	Minimum	Maximum
CCT (μm)	535.05 ± 35.96	444.00	609.33	532.84 ± 34.73	442.67	602.33
ACD (mm)	3.69 ± 0.22	3.18	4.28	3.79 ± 0.22	3.27	4.32
AQD (mm)	3.15 ± 0.28	2.52	3.93	3.26 ± 0.29	2.56	4.04
Km (D)	43.35 ± 1.31	40.21	46.83	43.35 ± 1.34	40.47	46.65
CD (mm)	11.65 ± 0.39	10.93	12.46	11.45 ± 0.37	10.64	12.31

*CCT* = central corneal thickness; *ACD* = anterior chamber depth; *AQD* = aqueous depth; *Km* = mean keratometry; *CD* = corneal diameter; *SD* = standard deviation

**Table 2:** The mean difference, standard deviation, *P* value and 95% limits of agreement (LoA) for differences between the SW-9000 and MS-39

Device pairings	Mean difference ± SD	*P* value	95% LoA
CCT (μm)	2.21 ± 2.67	< 0.001	− 3.01 to 7.44
ACD (mm)	− 0.10 ± 0.03	< 0.001	− 0.16 to − 0.05
AQD (mm)	− 0.10 ± 0.04	< 0.001	− 0.18 to − 0.03
Km (D)	− 0.01 ± 0.22	0.773	− 0.45 to 0.43
CD (mm)	0.20 ± 0.16	< 0.001	− 0.12 to 0.51

*CCT* = central corneal thickness; *ACD* = anterior chamber depth; *AQD* = aqueous depth; *Km* = mean keratometry; *CD* = corneal diameter; *SD* = standard deviation

**Fig. 1:**
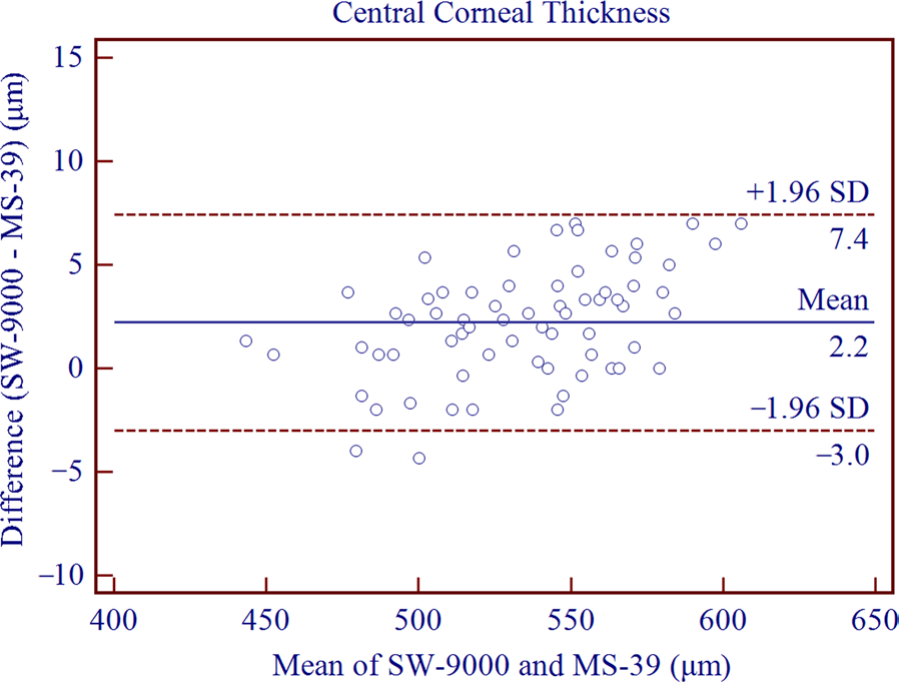
Bland-Altman plots of agreement for the center corneal thickness (CCT) measurement between SW-9000 and MS-39. The mean difference is indicated by the solid blue line, and the 95% LoA is denoted by the dashed red lines

**Fig. 2:**
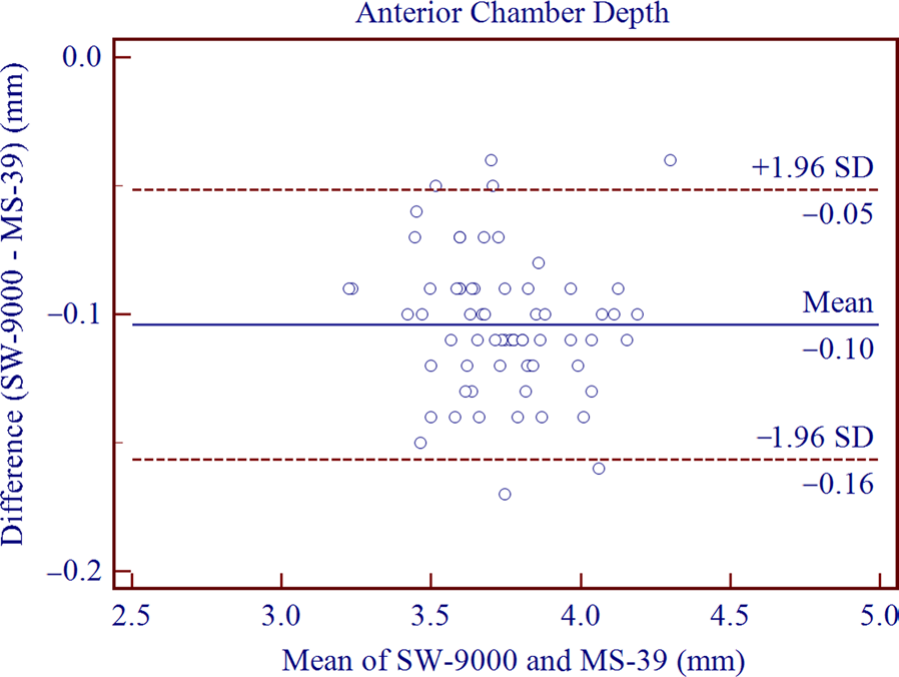
Bland-Altman plots of agreement for anterior chamber depth (ACD) between SW-9000 and MS-39. The mean difference is indicated by a solid blue line, and the 95% LoA is denoted by the dashed red lines

**Fig. 3:**
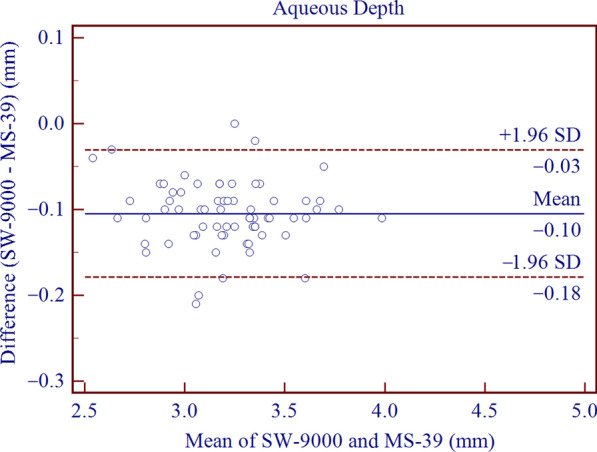
Bland-Altman plots of agreement for aqueous depth (AQD) between SW-9000 and MS-39. The mean difference is indicated by a solid blue line, and the 95% LoA is denoted by the dashed red lines

**Fig. 4:**
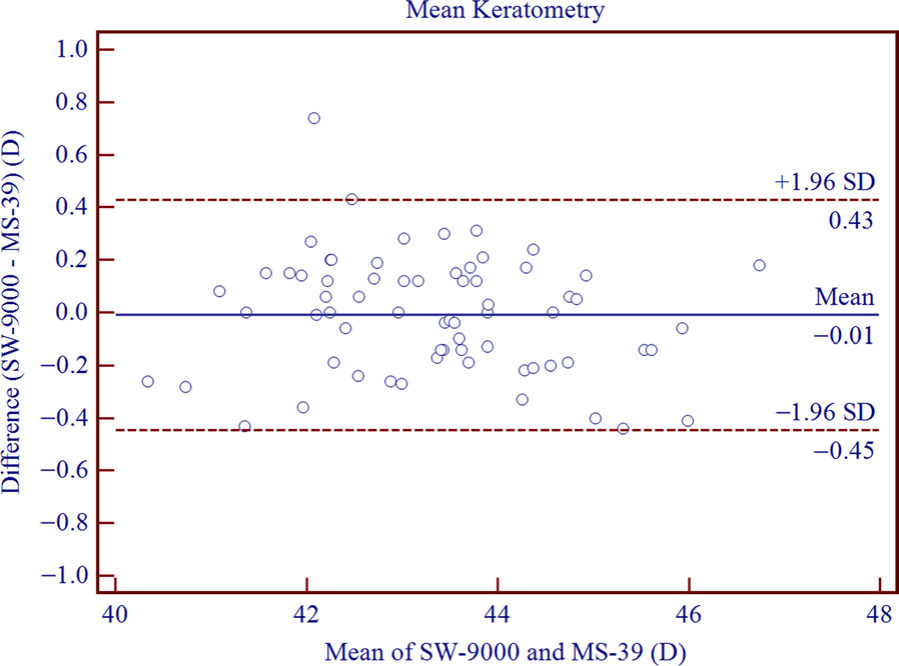
Bland-Altman plots of agreement for mean keratometry (Km) between SW-9000 and MS-39. The mean difference is indicated by a solid blue line, and the 95% LoA is shown by the dashed red lines

**Fig. 5:**
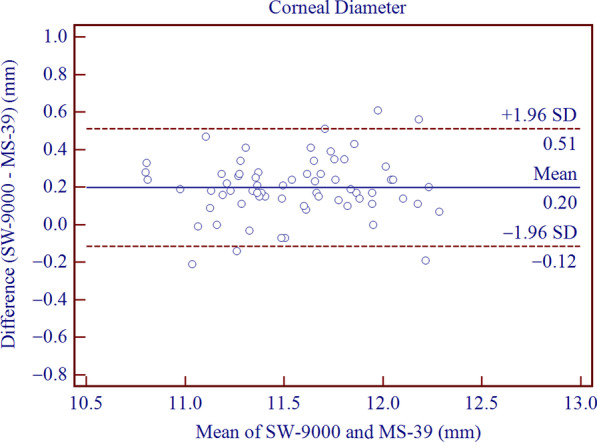
Bland-Altman plots of agreement for corneal diameter (CD) between SW-9000 and MS-39. The mean difference is represented by a solid blue line, and the 95% LoA is represented by the dashed red lines

Agreement analysis of Bland-Altman plots is shown in Figs. 1, 2, 3, 4, 5. When measuring CCT, ACD, AQD, Km and CD, the results showed high agreement between SW-9000 and MS-39, with narrow 95% LoA ranges.

## Discussion

Due to the growing importance of anterior segment measurements, numerous devices based on different principles have been developed. The most commonly used principles include standard corneal topography (with a Placido disk), Scheimpflug imaging, AS-OCT, color light-emitting diode (LED), OLCR as well as swept-source OCT (SS-OCT).

The SW-9000, a new OLCR-based optical biometer, has not yet been investigated. Any new device needs to be validated before it can be widely adopted in the clinical setting. It is considered eligible only if it is proven to be reliable and can be interchangeably used with other instruments. The MS-39 is the only device that combines SD-OCT and Placido. Previous studies have revealed high repeatability and reproducibility of MS-39 for anterior segment analysis [14, 19]. Good agreement was found when comparing the MS-39 with the Pentacam HR (OCULUS, Wetzlar, Germany) and Sirius (CSO, Florence, Italy), both of which are based on Scheimpflug imaging [14, 20, 21]. Similar results were obtained when investigating agreement between the MS-39 and SS-OCT-based devices such as Argos (Movu, Komaki, Japan) and ANTERION (Heidelberg, Heidelberg, Germany) [19, 22]. Besides, AS-OCT system like RTVue (Optovue, Fremont, CA) and PCI system like IOLMaster 500 (Carl Zeiss Meditec, Jena, Germany) had also been compared with MS-39. Nevertheless, no study has compared the MS-39 with the devices which adhered to OLCR principle. In order to comprehensively evaluate the accuracy of SW-9000, we compared the agreement and difference between the SW-9000 and MS-39.

When measuring CCT, our results revealed a significant difference between the MS-39 and SW-9000 (*P* < 0.001). The mean difference was 2.21 ± 2.67 μm, with the 95% LoA between − 3.01 and 7.44 μm. Comparison between Argos and MS-39 [19] showed that the mean difference was 5.78 ± 4.84 μm, and the 95% LoA was − 3.70 to 15.25 μm, nevertheless, the values in that study were larger than ours. Oh et al. [23] found excellent agreement between ANTERION based on SS-OCT and CASIA 2 (Tomey, Nagoya, Japan), with a mean difference value of 2.30 ± 6.30 μm and 95% LoA of − 10.06 to 14.65 μm. Hashemi et al. [24] compared an OLCR-based system (Lenstar LS900, Haag-Streit AG, Koeniz, Switzerland) and a Scheimpflug-Placido topographer (Pentacam HR) for measuring CCT, and obtained high agreement, where the mean difference was − 5.14 ± 7.52 μm, and 95% LoA was − 19.88 to 9.60 μm. The interval we obtained was narrower than those in most previous studies. Given the small mean difference value and narrow LoA in our study, we suggest that the two devices can be used interchangeably for CCT measurement.

In terms of ACD and AQD measurements, the SW-9000 presented lower values than the MS-39, as the mean difference were − 0.10 ± 0.03 mm and − 0.10 ± 0.04 mm, while the 95% LoAs were − 0.16 to − 0.05 mm and − 0.18 to − 0.03 mm, respectively. These intervals were slightly narrower than those reported by Ruan et al., who separately assessed agreement between the IOLMaster 700 (Carl Zeiss Meditec, Jena, Germany) and CASIA 2, yielding 95% LoAs of − 0.03 to 0.24 mm for ACD and 0.04 to 0.25 mm for AQD [25]. Similar results were reported when comparing the MS-39 and the Argos (− 0.01 ± 0.03 mm) for ACD and AQD measurements, which were insufficient to produce noticeable differences in clinic [19]. According to previous studies, the IOL power would change by 0.1 D with 0.1 to 0.2 mm change in ACD [26, 27]. Mean difference of 0.1 mm corresponds to a change of IOL power of 0.1 D, which has no influence in clinical practice. Consequently, although the differences obtained in our study were statistically significant, they were too small to have any noticeable impact on the refractive outcome.

As for the measurement of mean keratometry, the mean difference of − 0.01 ± 0.22 D indicated no significant difference between the two devices (*P* = 0.773), with 95% LoA was − 0.45 to 0.43 D. However, these results were slightly larger than those reported for the MS-39 and Argos [19]. Mehdizadeh et al. found that the calculated IOL power varies by 0.9 to 1.3 D for a 1.0 D change in K [28]. In addition, Jasvinder et al. reported that a difference of 1.0 D and 0.5 D in average K translates to approximately 1.0 D and 0.5 D difference in IOL power [29]. Hence, we strictly set the threshold of clinical difference at 0.5 D to ensure the visual acuity after IOL implantation. Under these circumstances, the absolute maximum value of the limit of 95% LoA (0.45 D) in this study was still less than the cutoff value. Apparently, when measuring Km, the difference between the two devices was clinically irrelevant.

CD, is an important parameter for determining the optical area in corneal refractive surgery and predict the vault after phakic IOL implantation. The mean difference in CD measurement between the MS-39 and SW-9000 was 0.20 ± 0.16 mm, with 95% LoA ranging between − 0.12 and 0.51 mm. When the MS-39 was compared to the Pentacam and Sirius, the 95% LoAs ranged between − 0.46 to + 0.19 mm and − 0.54 to + 0.47 mm, respectively, showing agreement close to the results obtained in a previous study [14]. Variations in detection methods as well as dissimilar methods of defining the limbus for various devices usually lead to non-optimal agreement [30–32]. Given that CD has been widely used for phakic IOL implantation and phakic IOLs are sized to the nearest 0.50 mm, ≥ 0.50 mm was set as the threshold for clinical difference [33, 34]. Therefore, 0.51 mm, which is a close approximation of the above threshold, indicates that the two instruments are interchangeable.

This study has some limitations. First, only young healthy people were included. Given the presence of multiple pathological eyes, such as keratoconus and post-corneal refractive surgery eyes, the good agreement between the two instruments in this study was not completely representative for different populations. Second, with increasing age, the corneal senile ring becomes more common, and whether this will worsen the agreement of CD measurement needs to be studied.

## Conclusion

When the subjects were healthy, the new OLCR biometer demonstrated high agreement with MS-39 in CCT, ACD, AQD, Km and CD measurements, suggesting that these two instruments could be used interchangeably in clinical practice.

## Data Availability

All data generated or analyzed during this study are included in this published article.

## Abbreviations

OLCR: Optical low coherence reflectometry
SD-OCT: Spectral-domain optical coherence tomographer
LoA: Limits of agreement
CCT: Central corneal thickness
ACD: Anterior chamber depth
AQD: Aqueous depth
Km: Mean keratometry
CD: Corneal diameter
IOL: Intraocular lens
AS-OCT: Anterior segment optical coherence tomographer
NCT: Non-contact tonometer
SLED: Superluminescent light emitting diode
PD: Pupil diameter
LED: Light-emitting diode
SS-OCT: Swept-source optical coherence tomography

## References

[1] Kim J, Eom Y, Yoon E, Choi Y, Song J, Jeong J (2022). Algorithmic intraocular lens power calculation formula selection by keratometry, anterior chamber depth and axial length. Acta Ophthalmol.

[2] Yin S, Guo C, Qiu K, Ng T, Li Y, Du Y (2022). Assessment of the influence of keratometry on intraocular lens calculation formulas in long axial length eyes. Int Ophthalmol.

[3] De Bernardo M, Cione F, Capasso L, Coppola A, Rosa N (2022). A formula to improve the reliability of optical axial length measurement in IOL power calculation. Sci Rep.

[4] Srivannaboon S, Chirapapaisan C, Chirapapaisan N, Lertsuwanroj B, Chongchareon M (2013). Accuracy of Holladay 2 formula using IOLMaster parameters in the absence of lens thickness value. Graefes Arch Clin Exp Ophthalmol.

[5] Sorkin N, Achiron A, Abumanhal M, Abulafia A, Cohen E, Gutfreund S (2022). Comparison of two new integrated SS-OCT tomography and biometry devices. J Cataract Refract Surg.

[6] Savini G, Taroni L, Hoffer K (2020). Recent developments in intraocular lens power calculation methods-update 2020. Ann Transl Med.

[7] Teshigawara T, Meguro A, Mizuki N (2018). Influence of pupil dilation on predicted postoperative refraction and recommended IOL to obtain target postoperative refraction calculated by using third- and fourth-generation calculation formulas. Clin Ophthalmol.

[8] Simon NC, Farooq AV, Zhang MH, Riaz KM (2020). The effect of pharmacological dilation on calculation of targeted and ideal IOL power using multivariable formulas. Ophthalmol Ther.

[9] Berjandy F, Nabovati P, Hashemi H, Yekta A, Ostadimoghaddam H, Sardari S (2021). Predicting initial base curve of the rigid contact lenses according to Javal keratometry findings in patients with keratoconus. Cont Lens Anterior Eye.

[10] Young G, Hall L, Sulley A, Osborn-Lorenz K, Wolffsohn JS (2017). Inter-relationship of soft contact lens diameter, base curve radius, and fit. Optom Vis Sci.

[11] Matsuda J, Hieda O, Kinoshita S (2008). Comparison of central corneal thickness measurements by Orbscan II and Pentacam after corneal refractive surgery. Jpn J Ophthalmol.

[12] Garcia Marin YF, Alonso-Caneiro D, Vincent SJ, Collins MJ (2022). Anterior segment optical coherence tomography (AS-OCT) image analysis methods and applications: a systematic review. Comput Biol Med.

[13] Morishige N, Magome K, Ueno A, Matsui TA, Nishida T (2019). Relations among corneal curvature, thickness, and volume in keratoconus as evaluated by anterior segment-optical coherence tomography. Invest Ophthalmol Vis Sci.

[14] Savini G, Schiano-Lomoriello D, Hoffer KJ (2018). Repeatability of automatic measurements by a new anterior segment optical coherence tomographer combined with Placido topography and agreement with 2 Scheimpflug cameras. J Cataract Refract Surg.

[15] Rosa N, Cione F, Pepe A, Musto S, De Bernardo M (2020). An advanced lens measurement approach (ALMA) in post refractive surgery IOL power calculation with unknown preoperative parameters. PLoS One.

[16] De Bernardo M, Borrelli M, Imparato R, Cione F, Rosa N (2020). Anterior chamber depth measurement before and after photorefractive keratectomy. Comparison between IOLMaster and Pentacam. Photodiagn Photodyn Ther..

[17] Lloyd McKernan A, O'Dwyer V, Simo ML (2014). The influence of soft contact lens wear and two weeks cessation of lens wear on corneal curvature. Cont Lens Anterior Eye.

[18] Bland JM, Altman DG (1986). Statistical methods for assessing agreement between two methods of clinical measurement. Lancet.

[19] Wang Q, Chen M, Ning R, Savini G, Wang Y, Zhang T (2021). The precision of a new anterior segment optical coherence tomographer and its comparison with a swept-source OCT-based optical biometer in patients with cataract. J Refract Surg.

[20] Vega-Estrada A, Mimouni M, Espla E, Alió Del Barrio J, Alio JL (2019). Corneal epithelial thickness intrasubject repeatability and its relation with visual limitation in keratoconus. Am J Ophthalmol.

[21] Schiano-Lomoriello D, Bono V, Abicca I, Savini G (2020). Repeatability of anterior segment measurements by optical coherence tomography combined with Placido disk corneal topography in eyes with keratoconus. Sci Rep.

[22] Schiano-Lomoriello D, Hoffer KJ, Abicca I, Savini G (2021). Repeatability of automated measurements by a new anterior segment optical coherence tomographer and biometer and agreement with standard devices. Sci Rep.

[23] Oh R, Oh JY, Choi HJ, Kim MK, Yoon CH (2021). Comparison of ocular biometric measurements in patients with cataract using three swept-source optical coherence tomography devices. BMC Ophthalmol.

[24] Hashemi H, Nabovati P, Khabazkhoob M, Emamian M, Yekta A, Fotouhi A (2020). Agreement of central corneal thickness measurements between Scheimpflug photography and optical low-coherence reflectometry in children. Semin Ophthalmol.

[25] Ruan X, Yang G, Xia Z, Zhang J, Gu X, Tan Y (2022). Agreement of anterior segment parameter measurements with CASIA 2 and IOLMaster 700. Front Med.

[26] Lackner B, Schmidinger G, Skorpik C (2005). Validity and repeatability of anterior chamber depth measurements with Pentacam and Orbscan. Optom Vis Sci.

[27] Utine CA, Altin F, Cakir H, Perente I (2009). Comparison of anterior chamber depth measurements taken with the Pentacam, Orbscan IIz and IOLMaster in myopic and emmetropic eyes. Acta Ophthalmol.

[28] Mehdizadeh M (2008). Effect of axial length and keratometry measurement error on intraocular lens implant power prediction formulas in pediatric patients. J AAPOS.

[29] Jasvinder S, Khang TF, Sarinder KK, Loo VP, Subrayan V (2011). Agreement analysis of LENSTAR with other techniques of biometry. Eye (Lond).

[30] Huang J, Savini G, Li J, Lu W, Wu F, Wang J (2014). Evaluation of a new optical biometry device for measurements of ocular components and its comparison with IOLMaster. Br J Ophthalmol.

[31] Namkung S, Boyle AB, Li Y, Gokul A, McGhee C, Ziaei M (2022). Repeatability and agreement of horizontal corneal diameter measurements between scanning-slit topography, dual rotating Scheimpflug camera with Placido disc tomography, Placido disc topography, and optical coherence tomography. Cornea.

[32] Güçlü H, Akaray İ, Kaya S, Sattarpanah S, Çınar A, Sakallıoğlu K (2021). Agreement of anterior segment parameters between schiempflug topography and swept-source optic coherence based optic biometry in keratoconus and healthy subjects. Eye Contact Lens.

[33] Tañá-Rivero P, Aguilar-Córcoles S, Rodríguez-Prats J, Montés-Micó R, Ruiz-Mesa R (2021). Agreement of white-to-white measurements with swept-source OCT, Scheimpflug and color LED devices. Int Ophthalmol.

[34] Salouti R, Nowroozzadeh MH, Zamani M, Ghoreyshi M, Khodaman AR (2013). Comparison of horizontal corneal diameter measurements using the Orbscan IIz and Pentacam HR systems. Cornea.

